# Study of the Load During Official Competition in Professional Women’s Basketball—A Case Study

**DOI:** 10.3390/sports13020059

**Published:** 2025-02-17

**Authors:** Pablo López-Sierra, Sergio L. Jiménez-Sáiz, Javier García-Rubio, María Isabel Piñar, Sergio J. Ibáñez

**Affiliations:** 1 Grupo de Optimización del Entrenamiento y el Rendimiento Deportivo (GOERD), Facultad de Ciencias del Deporte, Universidad de Extremadura, 10003 Caceres, Spain; pablols@unex.es (P.L.-S.); jagaru@unex.es (J.G.-R.); sibanez@unex.es (S.J.I.); 2 Sport Sciences Research Centre, Faculty of Education & Sport Sciences and Interdisciplinary Studies, Universidad Rey Juan Carlos, 28943 Fuenlabrada, Spain; 3 Structure and Processes Involved in Interaction Sports, Faculty of Sports Science, Universidad de Granada, 18011 Granada, Spain; maribelpinar@ugr.es

**Keywords:** woman, external load, internal load, specific positions, match outcome

## Abstract

**Background:** Basketball matches involve numerous conditioning factors which, when put together, make for a complex prediction event. However, unraveling all these elements in different studies allows the control of certain conditioning factors of the game, giving rise to more stable and controlled games. **Objectives:** Due to the absence of studies that analyze professional matches in women’s basketball, the aim of the present research is to analyze the influence of the result, partial result and specific positions on the load in official competition between two women’s teams that play a match simultaneously. **Methods:** Using an ex post facto design, 19 professional players were measured in an official match of the Spanish second division of women’s basketball, monitoring both opponents simultaneously, obtaining at all times the contrast of loads between both teams. Inertial devices were used to measure the external and internal load of each player. Parametric and non-parametric statistical difference analyses were performed, as well as two linear mixed models. **Results:** The results reveal significant differences in external loading when loads are analyzed as a function of match outcome. Differences between external and internal load are found when taking into account specific positions, and when including several comparisons in the analysis. The team that obtained the highest kinematic and neuromuscular load demands was the one that won the match. The specific position of guard obtained a greater physical load at the end of the match than the centers. **Conclusions:** Coaches should prioritize high-intensity training that replicates match demands, considering positional differences in workload to optimize player conditioning and recovery strategies for sustained performance.

## 1. Introduction

Basketball is an invasive team sport in which the exhaustive scheduling of players’ workloads is a challenge for physical trainers. A very useful tool for load control in competition is inertial devices (IMUs). Thanks to the use of these devices, it is possible to know objectively what the demands of basketball, in this case women’s basketball, are [1]. Regarding the objective internal load, professional female basketball players reach values above 190 beats per minute (bpm), maintaining an average of around 170 bpm throughout the game [2]. Regarding the external kinematic load, professional female basketball players perform during matches an average of 2.5 total kilometers [3], 237 m of explosive distance [3], around 14 m at sprint speed (>21 km/h) [3] and 1.76 jumps per minute on average. Women’s competition benchmark values for impacts are 1.65 impacts per minute [2] and an average player load of 0.98 au/min [3]. Some authors such as Ibáñez, et al. [4,5] carried out statistical analyses using data from inertial devices to determine the speed, acceleration and impact zones in men’s and women’s basketball according to level, thus allowing for greater individualization of training and facilitating the work of coaches.

However, the process of individualization by speed zones is the minimum level of depth, as it is only individualized to the sport practiced, basketball, and the category in which it is carried out, women’s professional basketball. For training to be optimal for performance in competition, the differences that appear between training and competition must be reduced [2,6]. These data can be taken from the competition in general, or by individualizing the data with the result of the match in mind.

Numerous scientific studies have compared the demands of competition, discriminating between winning and losing teams according to the final result; however, the vast majority of studies have been carried out in men’s basketball. Castillo, et al. [7] found significant differences in distance traveled between 12 and 24 km/h between winning and losing teams, in favor of the winning teams. However, the final result is not everything, as the partial result greatly influences the final outcome that may occur. Ramírez-Arroyo, et al. [8] conducted a study that looked at the influence of the partial score on the final result in men’s and women’s games. In women’s basketball, the team winning the second period won the game in 72.73% of the cases, followed by the first period winning in 72.08% of the games.

Considering the importance of the partial outcome of the periods in the final result, it seems logical to find literature on the physical demands of basketball as a function of the period. Pérez-Chao, et al. [9] analyzed the variation in loads depending on the periods in men’s basketball. Significant differences were found between the first and second period (distance, player load and slow-speed running), between the first and third period (distance, player load and high-speed distance running), between the first and last period (distance, player load and slow speed) and between the third and last period (distance and player load). Scanlan, et al. [10] added to their study the load of teams that had to extend the game with one or several overtimes. In the extensions, they found significant differences with respect to normal periods in the variables heart rate, sum of heart rate zones, low-intensity leftward direction changes, medium-intensity accelerations and medium-intensity leftward direction changes. All these values were higher in the extra time than in the regulation time.

Another factor to take into account when individualizing the loads of professional players is the specific positions they occupy on the field [11,12]. However, there are few studies that analyze the existing differences between the specific positions of the players, and these are mostly based on the analysis of performance and tactics. Studies such as the one by Pino-Ortega, et al. [13] analyzed the workload between specific positions in men’s basketball, finding significant differences related to distance, peak speed and peak acceleration, all in favor of point guards and with the lowest performance in pivots. The study by Reina, García-Rubio, Feu and Ibáñez [2], in women’s basketball, goes a step further and analyzes the differences between specific positions, also adding the differences that may arise between training and competition. In this case, there were differences between training and competition, in the case of player load and steps in all specific positions. In this study, the positions were divided into five roles (point guard, shooting guard, small forward, power forward and center). Another similar study was carried out by Garcia, et al. [14], analyzing the possible differences between the three main specific positions (point guard, small forward and center), also taking into account the period of play. In this case, differences were also found in the distance traveled, the distance traveled at high speed (>18 km/h) and the accelerations (>2 m/s^2^) and decelerations (<−2 m/s^2^) performed. However, one thing stands out from the latest study, and that is the novel analysis used to find the differences between the roles and the outcome.

Due to the absence of studies that analyze professional matches in women’s basketball, the aim of the present research is to analyze the influence of the result, partial result and specific positions on the load in official competition between two women’s teams that play a match simultaneously. It is hypothesized that there will be significant differences depending on the result in favor of the winner and between the most differential specific positions, guards and centers.

## 2. Materials and Methods

### 2.1. Design

This research was methodologically encompassed within the ex post facto designs, as quantitative data were analyzed descriptively and retrospectively [15]. The research was carried out in the natural context of sport, so the variables were not manipulated at any time.

This study employed a multivariate approach, incorporating multiple dependent and independent variables, a common practice in sports and physiological research. Such an approach is essential, as physiological and performance-related factors in sports are inherently interdependent and cannot be accurately assessed in isolation [16]. Given that match conditions are inherently non-replicable—each game presents unique contextual and situational factors—comprehensive analyses that consider multiple interacting variables are necessary to maximize the depth and applicability of the findings.

This is a single measurement study, analyzing two teams simultaneously in a single match. This affects the generalizability of the results, but in this case, it is considered of great relevance as it is a novel study whose great power is the analysis of two simultaneous opponents, leading to a greater understanding of why the load is produced in certain circumstances, being conditioned by the interaction between the two rivals, facilitating the knowledge about the origin of the load. Other studies analyze the load in competition in several matches, but in very different contexts, and the data are more dubious when it comes to generalizing. Despite being a larger sample size, there can be very different matches depending on the score, and by obtaining average values between very intense and very relaxed matches due to the difference in the score in both teams, when average values are analyzed statistically, the average smoothes out these opposite situations, finding data that are far from reality. The ideal would be to have a larger sample of matches in which both rivals are analyzed or, if only one rival is analyzed, at least analyze each match uniquely, and classify them by type of match, so as not to condition the results by the different contexts.

### 2.2. Participants

The participants of this research were 19 professional female players (age = 25.05 ± 4.612 years; height = 1.78 ± 0.09 m; weight = 70.89 ± 9.49 kg) belonging to two teams in the second-highest category of Spanish women’s basketball, Liga Femenina Challenge, during the 2021/22 season. The sample was chosen with a non-probabilistic convenience sample, due to the fact that data are normally only obtained from one of the two teams competing in a game for privacy and performance reasons. This is one of the few cases where the approval of two clubs is available to collect data from the opposing team simultaneously with the collection of data from the team under investigation, with the possibility of making comparisons relative to both. This makes the research novel by being able to make a complete study of what happens in two basketball teams simultaneously throughout a professional regular league game.

All team members were informed of the benefits and risks of participating in the research. An informed consent form was signed by all coaches, coaching staff and players of both teams. The research was conducted following the criteria of the Declaration of Helsinki (2013) and was approved by the Bioethics Committee of the University (234/2019).

#### Eligibility Criteria

The following criteria were used for the selection of participants: (i) having been monitored by the research group for at least one week during the season, (ii) having played at least three minutes continuously in the monitored match, (iii) not being injured or recovering from a long-term injury (>2 weeks) at the time of the match.

### 2.3. Measures

The two teams of an official regular league match of the Liga Femenina Challenge (Spanish second division) were monitored. Inertial devices with a sampling frequency of 100 Hz were used for each of the variables measured. The data were extracted by generating a database with a total of 65 cases, formed by the data of the periods played by each player of both teams (period 1 = 15 cases, period 2 = 16 cases, period 3 = 15 cases, period 4 = 19 cases).

The independent variables of the study were the result of the game (win or lose the game), the partial result of the game (win, draw or lose the period) and the specific positions of the players (point guard, forward, center).

As for the dependent variables, the total data for each variable were extracted and then normalized to the playing time, except for variables referring to maximum values, such as the maximum speed reached in the game. The variables extracted for analysis referred to time played, distance traveled, accelerations and decelerations, heart rate, speed, impacts received, jumps and player load. Table 1 shows each variable, with its unit of measurement and definition.

These teams were monitored for at least one training mesocycle during the same season. From these records, the maximum variables achieved are obtained to extract the relative values of the percentage variables.

### 2.4. Procedure

WIMU ProTM inertial devices (RealTrack Systems, Almeria, Spain) with eight ultra-wideband (UWB) antennas were used for indoor operation. The players were equipped with a harness with a pocket located at the level of the thoracic vertebrae T2-T4, being the optimal place for obtaining positioning data validated with these devices. In addition, a Garmin heart rate strap (Garmin Ltd., Olathe, KS, USA) was used for internal load monitoring.

The coaches and physical trainers of the two clubs were contacted to offer them the possibility of monitoring the league match between the two teams. After a favorable response from both, the data were collected at the match. For this purpose, on the day of the match, the eight UWB antennas were positioned opposite each other one hour before the match. During that hour, both teams were also equipped with harnesses with inertial devices and heart rate bands. A proprietary application of the inertial device system (SVIVO, RealTrack Systems, Almeria, Spain) was used to generate a timeline to differentiate each part of the match at the data analysis stage.

### 2.5. Statistical Analysis

After the match measurement, data extraction was performed with the inertial device analysis application (SPRO, RealTrack Systems, Almeria, Spain). For those variables requiring a cut-off threshold, such as speed, acceleration and deceleration, a k-means cluster analysis was performed, establishing five zones for the three clusters following the protocol of Ibáñez, López-Sierra, Lorenzo and Feu [5]. These values were applied for the variables sprint (using the center of the cluster of speed zone five) and high-speed running (using the lower cut-off of speed zone five). Regarding the acceleration zones, they were used to discriminate the maximum acceleration and deceleration values obtained in the match. With the extracted data, a database with the total values of each player and a database with the values for each period played was elaborated, including a total of fifteen continuous variables and five contextual variables (team, role, period, partial result and final result).

Statistical analysis was performed with the Jamovi 2.3.28 tool [17]. The criteria assumption tests were performed, grouping the variables into parametric and non-parametric. For parametric variables, significant differences between the means of each variable were calculated as a function of the final result using a *Student’s t-test*. For non-parametric variables, a *Mann–Whitney U test* was performed [18]. Next, an *ANOVA test* was performed for independent samples comparing the differences in each parametric variable according to the specific positions, and a *Krustal–Wallis one-factor non-parametric H-test* for those variables that did not meet the criteria assumption tests. The alpha level for significance was ρ ≤ 0.05. Pairwise comparisons were performed using the *Bonferroni post hoc test*. The effect size of the model was performed using the *partial eta squared* and the *effect size* of the pairwise comparisons was performed using *Cohen’s d* [19].

For repeated measures analysis, two *linear mixed models* were performed comparing variables in terms of final outcome and specific positions, and partial outcome, final outcome and specific positions. To analyze the model fits, the AIC/BIC values and the marginal and conditional R^2^ were considered. Also, for each analysis, the ICC values and their significance are presented in order to ascertain whether the random response of the subjects was significant. The *Bonferroni post hoc test* was used for pairwise comparisons.

The *effect size* for the *Student’s t-test* was measured with *Cohen’s d*, using the following ranges: low (0–0.2), moderate (0.2–0.6), high (0.6–1.2) and very high (>1.2). For the *Mann–Whitney U-test, the biserial rank correlation* was employed using the following ranges: trivial (0–0.1), small (0.1–0.3), moderate (0.3–0.5), high (0.5–0.7) and very high (0.7–0.9). In terms of *power*, a high power is considered when the value exceeds 0.80 [20].

## 3. Results

Table 2 shows the results of the differences in the mean values when comparing the variables as a function of the final result, using the hypothesis testing model depending on the nature of the data, parametric or non-parametric, respectively.

The results of the tests to compare the different variables according to the final result (win–lose) showed statistically significant differences in the variables Explosive distance, Maximum acceleration, High-intensity acceleration distance, Sprint, High-speed running, Maximum speed and Distance with high metabolic load (*p* < 0.05) in favor of the winning team. The effect size values were moderate for all variables, as was power, except for the variables High-Speed Running and High Metabolic Load Distance, whose values were classified as high in both effect size and statistical power.

Table 3 shows the results of the tests comparing the variables according to the specific positions of the players.

The results of the *ANOVA* and the *non-parametric Krustal–Wallis one-factor test* to compare the specific positions in each variable showed statistically significant differences in ten of the fifteen variables analyzed. Pairwise comparisons showed differences in various groups, mainly with forwards compared to guards and centers. Effect sizes were moderate in most cases, with high power (>0.80).

Table 4 presents the results of the *linear mixed model*, performed for the comparison of the final result (win vs. lose) and the specific positions (guard vs. forward vs. center). The *Bonferroni Post Hoc test* was used for pairwise comparisons.

The results of the *linear mixed model* showed improvements in the *marginal R*^2^ with respect to the *conditional R*^2^ for all variables, controlling for the random factor of individual subject response. However, the model is only significant for ten of the fifteen variables selected, which means that for five variables it would not be necessary to control for individual subject variability and it would be sufficient to take into account the specific position they occupy in the field. Most of the variables (ten of the fifteen analyzed) have a high *ICC*, suggesting that the individuality of the players has a significant effect on the results obtained. Four of these variables have an *ICC* > 0.7 (Maximum Heart Rate, Maximum Deceleration, Impacts and Player Load).

Significant differences were found between guards and centers in distance (*p* = 0.039); in the final result as a function of High Metabolic Load Distance (*p* = 0.047); between guards and centers according to the result in Explosive Distance (*p* = 0.028), High-Intensity Acceleration Distance (*p* = 0.033); and between guards and forwards according to the result with the variable High Metabolic Load Distance (*p* = 0.016).

Table 5 shows the results of the *linear mixed model*, comparing the partial result, the final outcome and the specific positions.

In this case, the results of the *linear mixed model* improve the *conditional R*^2^ obtained by the previous model, after adding the partial outcome of the match to the equation. However, despite being a better model, the number of significant variables with the mixed model remains the same.

Breaking down the results under each of the headings of the analysis carried out, significant differences were found between guards and centers in heart rate percentage (*p* = 0.038) and player load (*p* = 0.05) taking into account only specific positions, between guards and forwards in explosive distance (*p* = 0.008) taking into account the result, high-intensity acceleration distance (*p* = 0. 040), maximum speed (*p* = 0.033) and high metabolic load distance (*p* = 0.027) taking into account the result without specific positions; between the partial result and final outcome in impacts (*p* = 0.003) and player load (*p* = 0.016); and between guards and forwards considering the partial and final outcome together in maximum deceleration achieved (*p* = 0.011).

## 4. Discussion

The objective of the present research is to analyze the influence of the result, partial result and specific positions on the load in official competition between two women’s teams that play a match simultaneously. Differences were identified taking into account the final outcome of the match in external load (related to acceleration, speed and power); and between specific positions occupied in the field in internal and external load. Furthermore, differences were found in the comparisons between the three situations simultaneously (final result, partial result and specific positions), with a strong influence on the individuality of the subjects in the team.

The match analyzed can be defined as a typical balanced match, as the difference in the quality of the opposition was less than five places in the rankings. Matches between teams with more than five places difference are considered unbalanced, and more than ten, very unbalanced. The competition analyzed corresponds to the Spanish women’s professional second division. Ibáñez, Gómez-Carmona and Mancha-Triguero [4] identified small differences in the objective external load (speed, accelerations and impacts) between players in the first and second Spanish basketball divisions. The higher-level players have higher demands in these variables than the second-level players. However, as the differences are minimal, the second-division values can be used as a reference for first-division official competition matches, although these values may be exceeded in these leagues.

The literature currently contains very few published studies on official competition in professional women’s basketball. Research has been found that analyses competition in women’s professional teams [21,22,23]. The research carried out by Salazar, Castellano and Svilar [21] monitors a friendly competition, while the investigations of Roell, Helwig, Gollhofer and Roecker [22] and Sansone, Li, Confessore and Tessitore [23] analyze the official competition. All the research takes into account the load of the same team in different matches and, therefore, different contexts, each match being a unique case due to the specific and individual characteristics of the teams (quality, position in the ranking and style of play). This is why the results that can be found on the win–lose condition are highly conditioned by the quality of the opponent [24], the tactical differences of the opponents [25,26] and the moment of the match with respect to the score [27]. The research carried out is considered to be novel because it analyzes a match taking into account the results of two simultaneous opponents, in which the oscillation of the load will depend on the simultaneous interaction of both teams. The score is the same for both teams at all times, and the tactical situation is fed back from the problem solving that one team poses to its rival. Therefore, despite the great limitation of having monitored only one match to generalize the data, the present research provides the first reference data on the modulation of load in competition between two direct rivals in women’s basketball. More research should be undertaken in this line in the future, trying to monitor more games in which data from both teams are obtained and looking for how the tactics used influence the modulation of the load, as well as the final and partial results, and the score at each moment of the game.

The results of the tests to identify the differences in the external and internal load according to the result of the match, without controlling the individuality of each player, show significant differences in seven of the fifteen variables analyzed. The differences correspond to OCEL variables (Explosive Distance, Maximum Acceleration, High-Intensity Acceleration Distance, Sprint, High-Speed Running and Maximum Speed), in addition to the ONEL variable, High Metabolic Load Distance. It should be noted that all the variables with significant differences correspond to variables that represent high or maximum intensity, with the winning team’s players obtaining higher values. This result points to the importance of high-intensity training in professional women’s basketball [28] because players must be able to tolerate high rates of speed and acceleration in competition, and develop high power values [29]. Castillo, Raya-González, Clemente, Conte and Rodríguez-Fernández [7] analyzed competitive Full Game training tasks in 14 male professional players, without finding significant differences in the external load received by the players depending on the final result of the match. This is because these authors compared the result of Full Game tasks with a duration of 10 min, without taking into account the time accumulation of a real match. Therefore, these results are equivalent to the partial result of a match. Furthermore, it has been shown that the workload is not the same in a Full Game situation during training as it is during competition [2], so these results obtained during training should be analyzed with caution, as they do not reflect the demands of real competition. Training should prioritize developing players’ tolerance to high-intensity efforts like sprints, accelerations and high-speed runs. This requires specific exercises that mimic match demands, including high-intensity intervals, acceleration/deceleration work, and explosive strength–speed drills. Since Full Game tasks may not fully replicate competition demands, scheduling simulations with cumulative loads similar to a full match is essential for better preparation.

It has been identified that better physical condition is related to better sporting performance in both professional female basketball players [30] and junior players [31]. This largely depends on contextual factors such as the final result of a game, the moment of form or the moment of the season in which the players are competing [32]. Piñar, et al. [33] found that players who play more are the ones who accumulate more load, thus obtaining better performance values, because by playing more, they are more likely to generate positive performance actions for the team. This better assessment in turn affects the load because, according to the results of the study, the most-rested players are the ones who are able to develop more load in the competition. Zaric, et al. [34] highlight the importance of establishing statistical relationships between performance, fitness and technical aspects of the game to prepare players for competition. Studies such as those by Fort-Vanmeerhaeghe, Montalvo, Latinjak and Unnithan [31] and McGill, et al. [35], which relate concrete and specific conditional aspects of basketball with technical gestures that determine basketball performance, have begun to appear in the literature in order to work on tactics, technique and physical condition in a more logical and structured way, integrating all the aspects that condition performance in a single session. Training sessions should integrate physical, technical, and tactical development, emphasizing high-intensity basketball-specific movements like direction changes, shooting, and blocking to enhance decision making. Monitoring accumulated load is crucial, adjusting training based on the season and player condition to maintain performance in high-minute players and maximize readiness in others. This structured approach optimizes both individual and team performance.

The results of the tests for differences between the specific positions showed significant differences between the means of ten of the fifteen variables analyzed when comparing the specific positions (point guards, forwards and pivots). Few studies have analyzed the differences between the specific positions exclusively in official competition in women’s games. A similar study was carried out by Reina, García-Rubio, Feu and Ibáñez [2], in which they compared the specific positions in training (5vs5 tasks) and competition. However, the literature does contain several studies in men’s basketball that compare specific positions according to the external and internal demands of competition. The study by Fernández-Leo, et al. [36] found significant differences between specific positions in mean heart rate, player load and impacts. All variables coincide with the significant differences found in the present study. Similar results were obtained by Vázquez-Guerrero, et al. [37] in their study when analyzing twelve elite male players (men’s first division), finding differences in all variables related to acceleration. In this case, only those accelerations that were performed at high intensity coincide with the differences found in this study. The lack of studies in women’s basketball makes it unclear whether findings from men’s basketball can be used as a reference until new evidence emerges. Coaches should monitor players’ internal and external loads to understand their team’s competition demands and design training tasks that replicate those values.

The study by Coyne, Coutts, Newton and Haff [6] uses a linear mixed model to control for within-subject variability when comparing internal and external load in basketball. The researchers found that the variable player load was similar in training and competition in training before the Olympics. In this case, no relationship was found between the normalized player load variable and sporting performance, only when this variable was used using the Acute:Chronic Workload Ratio. However, this study did not take into account the specific positions of the players. Delextrat, et al. [38] in their study analyzed differences between specific positions, in this case, according to periods. The guards were the ones who performed the most diverse movements, but the centers were the ones who had to make the most intense efforts as the game progressed. This study analyzed the variability of the specific positions, which allows us to ascertain how the values of the players behave in each specific position; however, the analyses carried out do not allow us to have the necessary control of the intrasubject variability. The lack of studies accounting for individual differences in player comparisons underscores the need to adjust analysis methods in sports science, particularly in both men’s and women’s basketball.

When the model comparing the final outcome and the specific positions is fitted with the partial outcome, four of the ten significant variables in the above model see an increase in the ICC. However, the other six variables see a decrease in intra-subject variability. Despite looking like very similar models, since it was a very unbalanced match in which the winning team won in three of the four periods, not only does the ICC vary in some variables, but the significant differences are completely different. The only study in the literature that performs a similar linear mixed model in basketball is that of Garcia, Salazar and Fox [14] in which the periods and specific positions of several professional men’s games were analyzed. In that study, differences were found between guards and centers in decelerations and sprints, and between forwards and centers in accelerations and decelerations. The results are similar to those found in this study, as the differences are essentially between guards and forwards, both at the level of partial score and when comparing partial and final scores. Coaches should consider both total and partial scores when making player substitutions, as game progression and play periods impact positions differently.

The importance of the partial result is highlighted by Ramírez-Arroyo, Gómez-Carmona, García-Rubio and Ibañez [8] in their study analyzing the importance of the partial result on the final result, extracting percentages of the probability of success or defeat depending on how the match evolves. In addition to the partial or total result, Castillo, Raya-González, Clemente, Conte and Rodríguez-Fernández [7] highlight that, although many variables are influenced by the final result, high decelerations and jumps are conditioned by the defensive style of the team. These differences in load are reflected in the training tasks depending on the style of tasks that are selected, being different depending on the idea of the game that is to be played on the court on match day [25]. In the match analyzed, the style of play of both teams was very different. While the winning team had a dynamic style with continuous transitions, without stopping the movement of the ball, the losing team essentially used a positional attack. On the other hand, the winning team played a pressing individual defense in the full field, while the losing team played a midfield defense. These tactical differences inherently shaped movement patterns and intensity levels. A match played at a higher intensity—such as during a decisive tournament phase or against a more physically dominant opponent—could result in increased sprinting, decelerations and overall exertion. Conversely, a game with a slower pace, due to strategic choices or fatigue management, might yield lower physical demands. Additionally, different teams with varying tactical approaches could present unique load profiles, as a fast-paced team emphasizing counterattacks would experience different physical demands than a team focused on prolonged ball possession. Competition factors such as match stakes, crowd presence, or officiating styles could also alter the game’s intensity and physical demands. Therefore, when analyzing load differences, it is essential to account for not only the team’s tactical approach but also the broader context in which the match takes place.

The fact that the study is novel, being the first to monitor an official league match between two professional teams simultaneously, provides an opening for new studies that can complete the research objective presented here. In addition, both internal and external physical load demands, and their relationship with the result in competition, have been taken into account. The main limitation that can be found is that only a single match is monitored, as it is currently very utopian to measure more league matches, and even more so with the simultaneous monitoring of both direct rivals. This affects the generalizability of the data, although this is compensated by the interaction reflected in the load between the two rivals, as the monitoring was simultaneous. In addition, due to the complexity of obtaining data relating to high-level professional competition, it has not been possible to take into account other aspects that could have conditioned the players throughout the match, such as psychological aspects. As a future prospect, the quality of the opposition should be taken into account, taking as a reference the position of each team in the standings at the time of the match, as it can influence the outcome of the research. In addition, it is recommended to carry out analyses of the moment of the match (attack vs. defense) to analyze how the load modulates depending on the phase of the game in which the team is playing. If possible, for more complex studies, players could be paired if there was an individual defense, analyzing how the load affects them in pairs.

## 5. Conclusions

The results of this research have shown that there are differences in the load depending on the final result. The winning team presented higher kinematic and neuromuscular load demands. The players who play in the specific position of point guard have higher load demands during the competition in internal, external neuromuscular and external kinematic load variables than the pivots. These results are unique as data are not usually obtained for both teams participating in an official professional competition match.

Coaches and performance staff should prioritize high-intensity training sessions to replicate the physical demands observed in competition, ensuring players develop the capacity to sustain high-speed movements, accelerations and power outputs under match conditions. It should be taken into account that high-intensity actions represent a lower percentage compared to low-intensity actions, and this should be taken into account in the design of the sessions, giving each type of activity the time required. Given the positional variations identified, workload management should be adapted accordingly. Point guards, who experience higher neuromuscular and kinematic loads, require specific conditioning focused on endurance, agility and repeated high-intensity efforts, whereas pivots should emphasize explosive strength and reactive agility to meet the demands of their role.

The evolution of the match score plays a crucial role in shaping players’ physiological responses and movement patterns, affecting decision making regarding rotations, substitutions and tactical adjustments. Understanding how score margins influence exertion levels can help optimize player management, preventing performance decrements caused by fatigue accumulation. Furthermore, training environments should incorporate competitive stressors to better simulate the physiological and psychological demands of real-game scenarios. Strategies such as modifying score conditions, rewarding tactical success or creating high-pressure game situations can enhance players’ ability to perform under stress.

Finally, considering the significant influence of match outcomes on physiological load, post-match recovery protocols should be adjusted based on exertion levels to optimize subsequent training sessions. Load management should be periodized to account for accumulated fatigue, ensuring that players who experience higher match demands receive appropriate recovery while those with lower workloads maintain fitness levels. Implementing these findings in coaching and training methodologies will enhance player preparedness, contributing to improved performance and long-term athletic development.

## Figures and Tables

**Table 1:** Description of the dependent variables with their unit of measurement.

Variable	Unit of Measurement	Definition
Maximum Heart Rate	Beats per minute (bpm)	Number of heart contractions per unit of time
Maximum Heart Rate Percentage	Percentage (%)	Maximum Heart Rate Percentage achieved in the match compared to the historical Maximum Heart Rate obtained in the monitoring of the player
Distance	Meters (m)	Total distance covered
Explosive Distance	Meters (m)	Total distance covered with an acceleration greater than 1.12 m/s^2^
Maximum Acceleration	Meters per second squared (m/s^2^)	Maximum acceleration achieved
Maximum Deceleration	Meters per second squared (m/s^2^)	Maximum deceleration achieved
Distance covered with High-Intensity Acceleration	Meters (m)	Distance covered accelerating over 3 m/s^2^
Distance covered with High-Intensity Deceleration	Meters (m)	Distance covered decelerating over 3 m/s^2^
Sprint	Meters (m)	Distance traveled above the absolute sprint speed threshold (18.80 km/h)
High-Speed Running	Meters (m)	Distance covered running over 17 km/h.
Maximum Speed	Kilometers per hour (km/h)	Maximum Speed achieved
Impacts	Count (cont)	Total number of horizontal impacts
High-Intensity Impacts	Count (cont)	Number of horizontal impacts with a force greater than 8G
Player Load	Arbitrary Unit (au)	Player load based on the accumulation of accelerations in the three axes
High Metabolic Load Distance	Meters (m)	Distance covered by a player while her Metabolic Power is over 25.5 W/kg

**Table 2:** Differences between the averages depending on the final outcome of the match.

Variable Type	Variable	*n*	$\bar{x}$ ± *SD Winning*	$\bar{x}$ ± *SD Losing*	*Test (Std.)*	*Std*	*p*	*Test (ES)*	*ES*	*pow.*
OIL	Max. Heart Rate	65	186.00 ± 11.40	182.00 ± 13.60	b	520.00	0.92	r	0.02	0.06
	% Max. Heart Rate	65	84.10 ± 3.73	84.20 ± 6.42	b	401.00	0.10	r	0.24	0.25
OCEL	Distance	65	74.60 ± 4.55	73.60 ± 9.58	a	−0.20	0.84	d	−0.05	0.07
	Explosive Dist.	65	13.90 ± 2.41	12.00 ± 2.96	a	2.22	**0.03**	d	0.55	0.71
	Max. Acceleration	65	6.76 ± 1.18	5.76 ± 1.65	b	325.00	**0.01**	r	0.39	0.46
	Max. Deceleration	65	−6.96 ± 1.61	−6.09 ± 1.65	b	454.00	0.33	r	0.14	0.14
	Dist. with High Acceleration	65	7.81 ± 3.47	5.26 ± 2.38	b	310.00	**0.01**	r	0.41	0.50
	Dist. with High Deceleration	65	7.32 ± 3.10	5.80 ± 2.29	b	435.00	0.23	r	0.18	0.28
	Sprint	65	2.21 ± 0.63	1.34 ± 1.62	b	353.00	**0.02**	r	0.33	0.37
	High-Speed Running	65	5.31 ± 1.93	3.14 ± 2.07	a	2.86	**0.01**	d	0.70	0.87
	Maximum Speed	65	24.30 ± 2.99	21.10 ± 4.45	b	323.00	**0.01**	r	0.39	0.46
ONEL	Impacts	65	163.00 ± 11.30	139.00 ± 49.80	b	404.00	0.11	r	0.24	0.25
	High-Intensity Impacts	65	0.56 ± 0.30	0.58 ± 0.42	b	497.00	0.68	r	0.06	0.08
	Player Load	65	1.14 ± 0.28	0.97 ± 0.37	b	414.00	0.14	r	0.22	0.22
	High Metabolic Load Dist.	65	15.9 ± 3.28	12.6 ± 4.16	a	2.67	**0.01**	d	0.66	0.84

OIL: Objective Internal Load; OCEL: Objective Cinematic External Load; ONEL: Objective Neuromuscular External Load. a: *Student’s t*; b: *Mann–Whitney’s U*; d: *Cohen’s d*; r: *Biserial Range Correlation*. *Std*: Statistic; *ES*: Effect Size, *pow.*: Power.

**Table 3:** Results of *ANOVA tests* and *Krustal–Wallis one-factor test* comparing the specific positions.

Variable Type	Variable	*F*	*p*	*ES*	*pow.*	Post hoc	*t*	*p_Bonf_*
OIL	Max. Heart Rate	11.50	**<0.001**	0.565 ^b^	0.97	1–3	2.46	**0.048**
					0.97	1–5	4.74	**<0.001**
	% Max. Heart Rate	3.75	**0.033**	0.395 ^b^	0.98	1–5	2.33	0.651
OCEL	Distance	4.70	**0.013**	0.132 ^a^	0.95	1–5	3.06	**0.010**
	Explosive Distance	4.93	**0.010**	0.137 ^a^	0.95	3–5	3.11	**0.009**
	Max. Acceleration	1.06	0.357	0.204 ^b^	0.68			
	Max. Deceleration	2.02	0.146	0.230 ^b^	0.71			
	Dist. with High Acceleration	4.58	**0.017**	0.427 ^b^	0.91	1–5	2.79	**0.023**
	Dist. with High Deceleration	9.86	**<0.001**	0.644 ^b^	0.99	1–5	4.09	**<0.001**
					0.99	3–5	3.09	**0.012**
	Sprint	1.46	0.245	0.224 ^b^	0.71			
	High-Speed Running	0.08	0.925	0.050 ^b^	0.51			
	Max. Speed	2.36	0.107	0.220 ^b^	0.70			
ONEL	Impacts	3.27	**0.049**	0.624 ^b^	0.98	1–5	2.23	**0.088**
	High-Intensity Impacts	3.71	**0.033**	0.353 ^b^	0.85	1–3	2.33	**0.063**
					0.85	1–5	2.35	**0.061**
	Player Load	12.80	**<0.001**	0.604 ^b^	0.98	1–3	3.32	**0.006**
					0.98	1–5	4.43	**<0.001**
	High Metabolic Load Distance	5.02	**0.010**	0.139 ^a^	0.95	3–5	3.15	**0.007**

OIL: Objective Internal Load; OCEL: Objective Cinematic External Load; ONEL: Objective Neuromuscular External Load. ES: Effect Size. ^a^ = Partial eta-squared. ^b^ = Epsilon squared. *pow.*: power. Dif.: Differences. 1 = “Guard”, 3 = “Forward”, 5 = “Center”.

**Table 4:** Linear mixed model depending on the final result and specific positions.

Variable Type	Variable	*Marginal R* ^2^	*Conditional R* ^2^	*AIC*	*ICC*	*p*
OIL	Max. Heart Rate	0.184	0.819	473.044	0.778	**<0.001**
	% Max. Heart Rate	0.089	0.256	435.857	0.184	0.312
OCEL	Distance	0.139	0.235	479.454	0.111	0.504
	Explosive Distance	0.302	0.632	304.797	0.473	**0.003**
	Max. Acceleration	0.192	0.595	207.931	0.499	**<0.001**
	Max. Deceleration	0.141	0.774	205.498	0.736	**<0.001**
	Dist. with High Acceleration	0.338	0.742	304.495	0.610	**<0.001**
	Dist. with High Deceleration	0.263	0.707	298.599	0.602	**<0.001**
	Sprint	0.156	0.240	245.225	0.099	0.527
	High-Speed Running	0.150	0.503	311.368	0.415	**0.002**
	Max. Speed	0.247	0.354	349.243	0.142	0.328
ONEL	Impacts	0.155	0.809	628.413	0.774	**<0.001**
	High-Intensity Impacts	0.110	0.527	83.141	0.469	**<0.001**
	Player Load	0.229	0.853	2.772	0.809	**<0.001**
	High Metabolic Load Distance	0.336	0.506	362.646	0.256	0.106

OIL: Objective Internal Load; OCEL: Objective Cinematic External Load; ONEL: Objective Neuromuscular External Load.

**Table 5:** *Linear mixed model* based on partial outcome, final outcome and specific positions.

Variable Type	Variable	*Marginal R* ^2^	*Conditional R* ^2^	*AIC*	*ICC*	*p*
OIL	Max. Heart Rate	0.199	0.821	478.662	0.777	**<0.001**
	% Max. Heart Rate	0.154	0.274	441.310	0.142	0.421
OCEL	Distance	0.216	0.273	483.390	0.073	0.638
	Explosive Dist.	0.310	0.606	313.514	0.428	**0.009**
	Max. Acceleration	0.218	0.628	214.077	0.525	**<0.001**
	Max. Deceleration	0.175	0.802	205.395	0.760	**<0.001**
	Dist. with High Acceleration	0.333	0.717	315.153	0.575	**<0.001**
	Dist. with High Deceleration	0.274	0.708	306.185	0.598	**<0.001**
	Sprint	0.215	0.222	564.933	0.009	0.942
	High-Speed Running	0.150	0.470	322.060	0.377	**0.005**
	Max. Speed	0.251	0.336	358.935	0.114	0.437
ONEL	Impacts	0.184	0.828	628.878	0.790	**<0.001**
	High-Intensity Impacts	0.122	0.503	92.714	0.435	**0.002**
	Player Load	0.247	0.858	6.449	0.811	**<0.001**
	High Metabolic Load Distance	0.329	0.465	373.099	0.203	0.200

OIL: Objective Internal Load; OCEL: Objective Cinematic External Load; ONEL: Objective Neuromuscular External Load.

## Data Availability

Data are unavailable due to privacy restrictions.

## References

[1] Power C.J., Fox J.L., Dalbo V.J., Scanlan A.T. (2022). External and Internal Load Variables Encountered During Training and Games in Female Basketball Players According to Playing Level and Playing Position: A Systematic Review. Sports Med. Open.

[2] Reina M., García-Rubio J., Feu S., Ibáñez S.J. (2019). Training and Competition Load Monitoring and Analysis of Women’s Amateur Basketball by Playing Position: Approach Study. Front. Psychol..

[3] Portes R., Jiménez S.L., Navarro R.M., Scanlan A.T., Gómez M.A. (2020). Comparing the External Loads Encountered during Competition between Elite, Junior Male and Female Basketball Players. Int. J. Environ. Res. Public. Health.

[4] Ibáñez S.J., Gómez-Carmona C.D., Mancha-Triguero D. (2022). Individualization of Intensity Thresholds on External Workload Demands in Women’s Basketball by K-Means Clustering: Differences Based on the Competitive Level. Sensors.

[5] Ibáñez S.J., López-Sierra P., Lorenzo A., Feu S. (2023). Kinematic and Neuromuscular Ranges of External Loading in Professional Basketball Players during Competition. Appl. Sci..

[6] Coyne J.O.C., Coutts A.J., Newton R.U., Haff G.G. (2021). Relationships Between Different Internal and External Training Load Variables and Elite International Women’s Basketball Performance. Int. J. Sports Physiol. Perform..

[7] Castillo D., Raya-González J., Clemente F.M., Conte D., Rodríguez-Fernández A. (2021). The effects of defensive style and final game outcome on the external training load of professional basketball players. Biol. Sport..

[8] Ramírez-Arroyo A.P., Gómez-Carmona C.D., García-Rubio J., Ibañez S.J. (2022). Influence of Match Status in Match Outcome of the Game in Professonal Basketball regarding Sex and Type of Competition. Ricyde-Rev. Int. Cienc. Deporte.

[9] Pérez-Chao E.A., Gómez M.A., Scanlan A., Ribas C., Trapero J., Lorenzo A. (2023). Influence of game and quarter results on external peak demands during games in under-18 years, male basketball players. Biol. Sport..

[10] Scanlan A.T., Stanton R., Sargent C., O’Grady C., Lastella M., Fox J.L. (2019). Working Overtime: The Effects of Overtime Periods on Game Demands in Basketball Players. Int. J. Sports Physiol. Perform..

[11] Fernández-Cortés J.A., Mandly M.G., García-Rubio J., Ibáñez S.J. (2021). Contribution of professional basketball players according to the specific position and the competition phase. E-Balonmano Com..

[12] Bordón J.C.P., Bravo I.R., Gajardo M.A.L., García J.D. (2021). Training load monitoring by position and task in professional men’s basketball. E-Balonmano Com..

[13] Pino-Ortega J., Rojas-Valverde D., Gómez-Carmona C.D., Bastida-Castillo A., Hernández-Belmonte A., García-Rubio J., Nakamura F.Y., Ibáñez S.J. (2019). Impact of Contextual Factors on External Load During a CongestedFixture Tournament in Elite U’18 Basketball Players. Front. Psychol..

[14] Garcia F., Salazar H., Fox J.L. (2022). Differences in the Most Demanding Scenarios of Basketball Match-Play Between Game Quarters and Playing Positions in Professional Players. Montenegrin J. Sports Sci. Med..

[15] Thomas J., Nelson J., Silverman S. (2015). Research Methods in Physical Activity.

[16] Biddle S.J.H., Markland D., Gilbourne D., Chatzisarantis N.L.D., Sparkes A.C. (2001). Research methods in sport and exercise psychology: Quantitative and qualitative issues. J. Sports Sci..

[17] The_jamovi_Project, Version 2.3; [Computer Software]; jamovi. https://www.jamovi.org.

[18] Newell J., Aitchison T., Grant S. (2010). Statistics for Sports and Exercise Science.

[19] Field A. (2013). Discovering Statistics Using SPSS.

[20] O’Donoghue P. (2012). Statistics for Sport and Exercise Studies. An Introduction.

[21] Salazar H., Castellano J., Svilar L. (2020). Differences in External Load Variables Between Playing Positions in Elite Basketball Match-Play. J. Hum. Kinet..

[22] Roell M., Helwig J., Gollhofer A., Roecker K. (2020). Duration-Specific Peak Acceleration Demands During Professional Female Basketball Matches. Front. Sports Act. Living.

[23] Sansone P., Li F., Confessore E., Tessitore A. (2024). Monitoring training load and perceived recovery indicators during the preseason and in-season phases in professional female basketball players. J. Sports Med. Phys. Fit..

[24] Sampaio J., Lago C., Casais L., Leite N. (2010). Effects of starting score-line, game location, and quality of opposition in basketball quarter score. Eur. J. Sport. Sci..

[25] Scanlan A.T., Wen N., Tucker P.S., Borges N.R., Dalbo V.J. (2014). Training Mode’s Influence on the Relationships Between Training-Load Models During Basketball Conditioning. Int. J. Sports Physiol. Perform..

[26] Qarouach A., Sansone P., Pernigoni M., Kreivyte R., Conte D. (2024). Inside the Defensive Playbook: Pick-and-Roll Tactical Adjustments Impact the External and Internal Loads During Small-Sided Games in Female Basketball Players. Int. J. Sports Physiol. Perform..

[27] Qiu M.J., Zhang S.L., Yi Q., Zhou C.J., Zhang M.X. (2024). The influence of “momentum” on the game outcome while controlling for game types in basketball. Front. Psychol..

[28] Haghighi A.H., Hosseini S.B., Askari R., Shahrabadi H., Ramirez-Campillo R. (2024). Effects of plyometric compared to high-intensity interval training on youth female basketball player’s athletic performance. Sport. Sci. Health.

[29] Aschendorf P.F., Zinner C., Delextrat A., Engelmeyer E., Mester J. (2019). Effects of basketball-specific high-intensity interval training on aerobic performance and physical capacities in youth female basketball players. Physician Sportsmed..

[30] Ibáñez S.J., Pinar M.I., García D., Mancha-Triguero D. (2023). Physical Fitness as a Predictor of Performance during Competition in Professional Women’s Basketball Players. Int. J. Environ. Res. Public. Health.

[31] Fort-Vanmeerhaeghe A., Montalvo A., Latinjak A., Unnithan V. (2016). Physical Characteristics of Elite Adolescent Female Basketball Players and Their Relationship to Match Performance. J. Hum. Kinet..

[32] García D., Escobar R., Pinar M.I. (2021). Effect of Situational Factors on the Rate of Perceived Exertion of Liga Femenina 2 Players. Rev. Psicol. Deporte.

[33] Piñar M.I., García D., Mancha-Triguero D., Ibáñez S.J. (2022). Effect of Situational and Individual Factors on Training Load and Game Performance in Liga Femenina 2 Basketball Female Players. Appl. Sci..

[34] Zaric I., Dopsaj M., Markovic M. (2018). Match performance in young female basketball players: Relationship with laboratory and field tests. Int. J. Perform. Anal. Sport..

[35] McGill S.M., Andersen J.T., Horne A.D. (2012). Predicting Performance and Injury Resilience from Movement Quality and Fitness Scores in a Basketball Team over 2 Years. J. Strength. Cond. Res..

[36] Fernández-Leo A., Gómez-Carmona C.D., García-Rubio J., Ibáñez S.J. (2020). Influence of Contextual Variables on Physical and Technical Performance in Male Amateur Basketball: A Case Study. Int. J. Environ. Res. Public. Health.

[37] Vázquez-Guerrero J., Suarez-Arrones L., Casamichana D., Rodas G. (2018). Comparing external total load, acceleration and deceleration outputs in elite basketball players across positions during match play. Kinesiology.

[38] Delextrat A., Badiella A., Saavedra V., Matthew D., Schelling X., Torres-Ronda L. (2017). Match activity demands of elite Spanish female basketball players by playing position. Int. J. Perform. Anal. Sport..

